# Brief Mental Training Reorganizes Large-Scale Brain Networks

**DOI:** 10.3389/fnsys.2017.00006

**Published:** 2017-02-28

**Authors:** Yi-Yuan Tang, Yan Tang, Rongxiang Tang, Jarrod A. Lewis-Peacock

**Affiliations:** ^1^Department of Psychological Sciences, Texas Tech UniversityLubbock, TX, USA; ^2^Department of Psychological and Brain Sciences, Washington University in St. LouisSt. Louis, MO, USA; ^3^Department of Psychology, University of Texas at AustinAustin, TX, USA

**Keywords:** integrative body–mind training (IBMT), multivariate pattern analysis (MVPA), resting-state fMRI, functional connectivity, large-scale brain networks

## Abstract

Emerging evidences have shown that one form of mental training—mindfulness meditation, can improve attention, emotion regulation and cognitive performance through changing brain activity and structural connectivity. However, whether and how the short-term mindfulness meditation alters large-scale brain networks are not well understood. Here, we applied a novel data-driven technique, the multivariate pattern analysis (MVPA) to resting-state fMRI (rsfMRI) data to identify changes in brain activity patterns and assess the neural mechanisms induced by a brief mindfulness training—integrative body–mind training (IBMT), which was previously reported in our series of randomized studies. Whole brain rsfMRI was performed on an undergraduate group who received 2 weeks of IBMT with 30 min per session (5 h training in total). Classifiers were trained on measures of functional connectivity in this fMRI data, and they were able to reliably differentiate (with 72% accuracy) patterns of connectivity from before vs. after the IBMT training. After training, an increase in positive functional connections (60 connections) were detected, primarily involving bilateral superior/middle occipital gyrus, bilateral frontale operculum, bilateral superior temporal gyrus, right superior temporal pole, bilateral insula, caudate and cerebellum. These results suggest that brief mental training alters the functional connectivity of large-scale brain networks at rest that may involve a portion of the neural circuitry supporting attention, cognitive and affective processing, awareness and sensory integration and reward processing.

## Introduction

Mindfulness meditation is one form of mental training methods including several key components, such as body relaxation, breathing practice, mental imagery and mindfulness practice (Tang et al., 2015a; Acevedo et al., 2016), and has been reported to reduce stress, improve attention, emotion regulation and cognitive performance (Tang et al., 2007). The integrative body–mind training (IBMT; or simply integrative meditation) is one form of mindfulness meditation originated from ancient eastern contemplative traditions and includes techniques of body relaxation, mental imagery and mindfulness guided by an IBMT coach. Cooperation between the body and the mind is emphasized in facilitating and achieving a meditative state. The trainees concentrated on achieving a balanced state of body and mind. The method stresses no effort to control thoughts, but instead a state of restful alertness that allows a high degree of awareness of body, mind, and external instructions (Tang et al., 2007, 2010, 2012). Our previous randomized studies have shown that short-term IBMT can improve attention, emotion regulation and cognitive performance through changing brain activity and white matter structural connectivity (Tang et al., 2007, 2009, 2010, 2012, 2013, 2015a,b). However, whether and how IBMT alters large-scale brain networks remains unknown.

The resting-state fMRI (rsfMRI) measures spontaneous neuronal activity of the brain and has been proven as an effective method for measuring large-scale functional networks in neuropsychology conditions. Therefore rsfMRI may be helpful for exploring the network alterations induced by short-term IBMT (Fox and Raichle, 2007; Tang et al., 2009, 2013).

Multivariate pattern analysis (MVPA) is a novel data-driven technique (Haynes and Rees, 2006; Norman et al., 2006; Pereira et al., 2009; Tong and Pratte, 2012; Lewis-Peacock and Norman, 2013, 2014) and has been paid increasing attention in rsfMRI analysis (De Martino et al., 2008; Haxby, 2012). MVPA has been applied in cognitive processing, brain aging, and mental disorders such as depression, antisocial personality disorder, attention-deficit disorder and schizophrenia (Dosenbach et al., 2010; Shen et al., 2010; Lewis-Peacock et al., 2012; Zeng et al., 2012). Studies suggested that MVPA could potentially detect spatially distributed information to further highlight the neural mechanisms underlying the behavioral symptoms (Zeng et al., 2012). Furthermore, MVPA based on whole-brain rsfMRI data can complement seed-based analyses. The whole-brain functional connectivity, unlike those analyzing several predefined regions or networks of interest, can ensure the optimal use of the wealth of information present in the brain imaging data (Zeng et al., 2012).

Hence, by using MVPA, our study employed whole-brain rsfMRI data to investigate the significant training-induced brain pattern changes in an undergraduate group who received 2 weeks of IBMT with 30 min per session for 10 sessions (5 h training in total). We hypothesize that the altered functional connections will be observed in the large-scale whole-brain resting-state networks including areas associated with attention, cognitive and emotional processing, awareness and sensory integration, and reward processing (Tang et al., 2007, 2009, 2010, 2012, 2013, 2015a,b; Acevedo et al., 2016). This exploration will be helpful in further discovering the neural mechanisms underlying the altered brain states, and may offer additional information for advancing our understanding of meditation training.

## Materials and Methods

### Participants

Twenty-five (13 males, 21 ± 1.6 years old) healthy undergraduates at Dalian University of Technology (DUT) without any meditation experience were recruited and completed 2 weeks of IBMT training with 30 min per session for 10 sessions (5 h training in total). This study was carried out in accordance with the recommendations of DUT Institutional Review committee. All subjects gave written informed consent in accordance with the Declaration of Helsinki. The protocol was approved by the DUT Institutional Review committee.

### Data Acquisition

Imaging data collection was performed with a Philips-Achieva 3T scanner (Eindhoven, Netherlands) at Dalian Municipal Central Hospital. During the experiments, the subjects were instructed to relax, and lie still with eyes focused on a central white cross on a black screen during the resting scan. Foam pads with a standard birdcage head coil were used to fix the subject’s head (Tang et al., 2013). Functional images were acquired using a gradient-echo EPI sequence (TR = 2000 ms, TE = 30 ms, flip angle = 80°). Whole-brain volumes were acquired with 36 contiguous 4-mm-thick transverse slices without gap. Functional resting-state session lasted 6 min and 10 s, and 180 volumes were obtained. For each subject, we collected the data before and after training.

### Preprocessing

All resting-state images were pre-processed using the SPM8 package (Wellcome Trust Center for Neuroimaging, University College London, London, UK^1^) and Data Processing Assistant for Resting-State fMRI (DPARSF)^2^. For each subject, the first five volumes of the scanned data were discarded due to magnetic saturation. The remaining volumes were corrected for within-scan acquisition time differences between slices, and realigned to the first volume to correct for inter-scan head motions. All subjects in this study had less than 1.5 mm translation in the *x*, *y*, or *z*-axes and less than 1.5° of rotation in each axis. Next, the volumes were normalized to a standard echo planar imaging template in the Montreal Neurological Institute (MNI) space. Then, smoothing and filtering were performed using a Gaussian filter of 8 mm full-width half-maximum kernel and a Chebyshev band-pass filter (0.01–0.08 Hz) respectively. Considering several potential sources of physiological noise in the functional connectivity analysis, nuisance covariates including head motion parameters, global mean signals, white matter signals and cerebrospinal fluid signals were regressed out from the image (Dosenbach et al., 2010).

The processed images were divided into 116 regions according to the automated anatomical labeling (AAL) atlas (Schmahmann et al., 1999). Regional mean time series were obtained for each subject by averaging the fMRI time series over all the voxels in each of the 116 regions (Shen et al., 2010). Pearson’s correlation coefficients were used to evaluate functional connectivity between each pair of regions and we obtained a resting-state functional network that was expressed as a 116 × 116 symmetrical matrix for each subject. By removing the 116 diagonal elements, the 6670 upper triangular elements of the functional connectivity matrix were normalized using Fisher’s *z*-transform, and were then used as the features in the subsequent MVPA.

### Features with High Discriminative Power

Reducing the number of features in a pattern classification problem can diminish noise, reduce overfitting and accelerate computation. In our analysis, feature selection reconstructs the feature space for classification by retaining the most discriminating functional connections and eliminating the rest. The discriminative power of a feature can be quantitatively measured by its relevance to classification (Guyon and Elisseeff, 2003). Therefore, the highly discriminating functional connections principally represented the alterative resting-state functional connectivity patterns. We can use these connections, rather than the full set of 6670 functional connections, to classify different brain states in the rsfMRI data before vs. after IBMT training.

In this study, we used the Kendall tau rank correlation coefficient (Kendall and Gibbons, 1990; Shen et al., 2010; Zeng et al., 2012), which provides a distribution-free test of independence between two variables to measure the relevance of each feature for classification. Suppose that there are *n* samples in the subjects after 2 weeks of IBMT. Let *x_ij_* denotes the functional connectivity feature *i* of the *j*th sample and *y_j_* denotes the class label of this sample (+1 for “post-training” and −1 for “pre-training”). The Kendall tau correlation coefficient of the functional connectivity feature *i* can be defined as:

(1) $$\tau_{i}\;=\;\frac{n_{c}-n_{d}}{n^{2}}$$

Where *n_c_* and *n_d_* are the number of concordant and discordant pairs, respectively. Because we do not consider the relationship of two samples, the total number of sample pairs is *n*^2^. For a pair of observation datasets {*x_ij_y_j_*} and {*x_ik_y_k_*}, it is a concordant pair when

(2) $$sgn(x_{ij}-x_{ik})\;=\;sgn(y_{j}-y_{k})$$

Correspondingly, it is a discordant pair when

(3) $$sgn(x_{ij}-x_{ik})\;=\;-sgn(y_{j}-y_{k})$$

**Figure 1:**
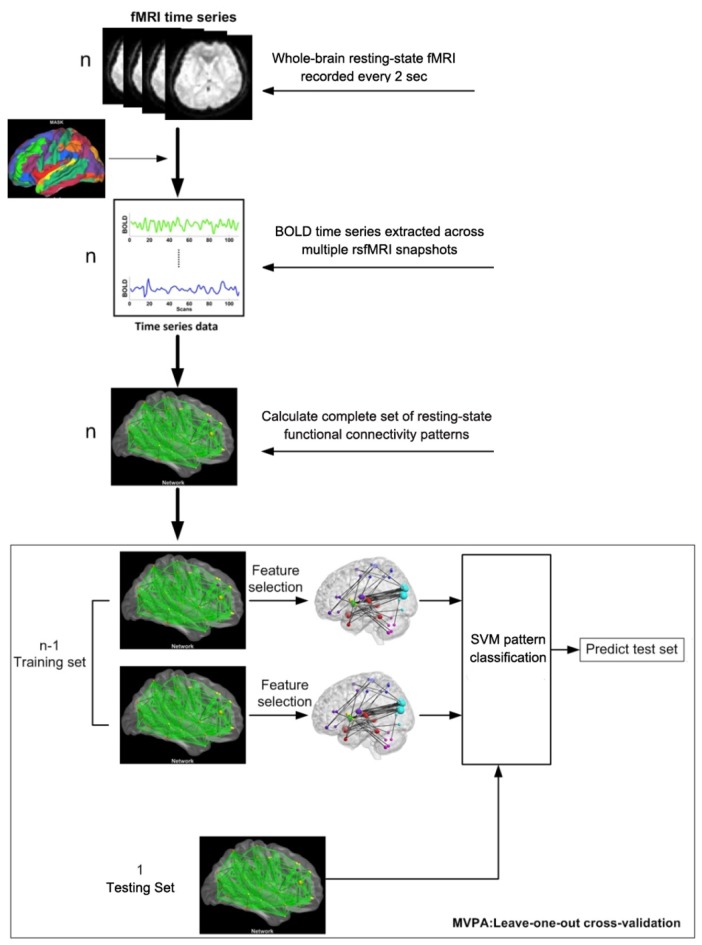
**Flow chart of the multivariate pattern analysis (MVPA) algorithm**.

Thus, a positive correlation coefficient *τ_i_* represents the *i*th functional connectivity feature that exhibits a significant increase after IBMT training, while a negative correlation coefficient *τ_i_* represents the *i*th functional connectivity feature that exhibits a significant decrease after training. We defined the “discriminative power” of a given feature as the absolute value of its Kendall tau correlation coefficient. When the absolute value of *τ_i_* was larger, the discriminative power was stronger. We ranked every *τ_i_* according to its discriminative power and then selected those features with scores above a certain threshold as the final feature set for classification. Because a leave-one-out cross-validation strategy was used to test the generalizability of the classifier (Figure 1), the final feature sets differed slightly across iterations of the classification procedure. Cross-validation ensures that the classifier is trained on tested on independent data, thus avoiding concerns of double-dipping or circularity in the classification results (Kriegeskorte et al., 2009). Next, we defined the “consensus functional connectivity” as the functional connectivity features that appeared (i.e., showed sufficiently strong discriminative power) in every cross-validation iteration (Dosenbach et al., 2010; Zeng et al., 2012). Finally, we calculated the “region weight” of each feature by counting the number of times that feature appeared in the consensus functional connections in this study. Region weights represented the relative contribution of each feature to the classifier’s discrimination of functional connectivity patterns in the rsfMRI data before vs. after IBMT training.

### Support Vector Classification and Permutation Tests

After obtaining the data set of features with high discriminative power, we used support vector machines (SVM) with radial basis kernel function to perform the classification. The kernel function we used was:

(4) $$k(x_{i},x_{j})\;=\;\exp\left(\frac{-{\left\|x_{i}-x_{j}\right\|}^{2}}{2\sigma^{2}}\right)$$

Here, sigma equaled 2. Due to our limited number of samples, we used a leave-one-out cross-validation strategy to estimate the performance of our classifier. Classification performance can be quantified using the generalization rate (GR), sensitivity and specificity based on the results of cross-validation. Note that the sensitivity represents the proportion of “post-training” samples correctly identified, while the specificity represents the proportion of “pre-training” samples correctly identified. The overall proportion of samples correctly predicted defines the GR.

Permutation tests were conducted to assess the performance of the classifier. In this study, the GR was chosen as the statistic to estimate the statistical significance of the classifier’s performance. For each classification iteration, we randomly permuted 1000× the class labels (“pre-training” or “post-training”) of the data being used to train the classifier. Importantly, the entire classification operation, including the feature selection and SVM, was carried out on every set of randomized class labels. We defined the GR as the performance of the classifier trained on permuted class labels, and we defined GR0 as the performance of the classifier trained on valid class labels. The *p*-values reported for classifier performance represent the probability of GR being no less than GR0. Therefore, when *p* < 0.05, this would indicate that the classifier could reliably decode whether the functional connectivity data was a pre-training or post-training sample.

### Reliability of the Algorithm

Recent attention has focused on the possibility for systematic bias in fMRI scans resulting from in-scanner motion (Satterthwaite et al., 2013). As the optimal procedures for removing motion artifacts are still an ongoing area of research, and it is unclear exactly how different methods impact downstream analyses, we chose to test our main hypotheses on motion-corrected (“scrubbed”) data. We implemented a scrubbing procedure as part of fMRI preprocessing. An estimate of motion at each time point was calculated as the frame-wise displacement (FD), using the three translational and three rotational displacements from rigid body motion correction procedure. Rotational displacements were converted from degrees to millimeters by calculating displacement on the surface of a sphere of radius 50 mm. Any frame *i* with *FD_i_* > 0.5 mm was linearly interpolated. We found there was no material difference in the results obtained from scrubbed vs. unscrubbed data, confirming the reliability of our algorithm.

## Results

### Classification Results

**Figure 2:**
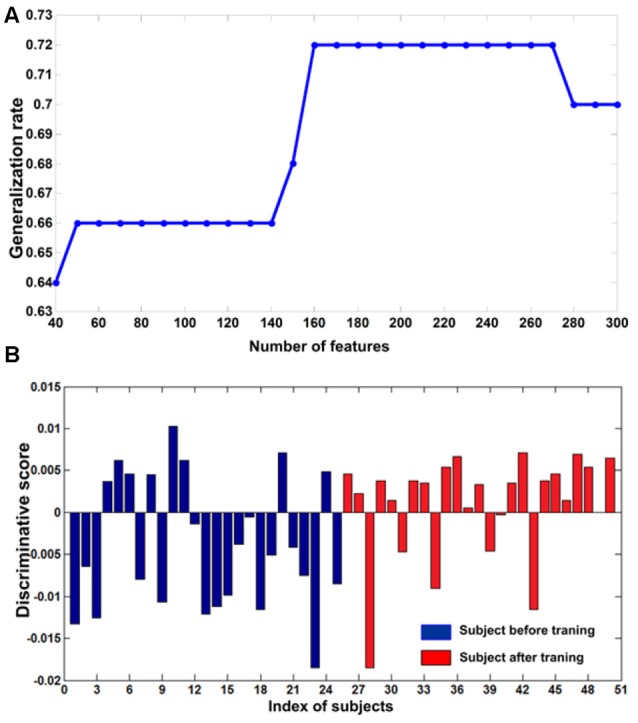
**(A)** The curve of the generalization rate (GR) to the number of features. The horizontal axis represents the number of selected features and the vertical axis represents the GR. **(B)** The discriminative scores of all subjects. The first 25 samples represented subjects before training (blue bar). The remaining samples represented corresponding subjects after training (red bar).

To estimate the effect of the selected parameters on the performance of the classifier, the cross-validation calculation was explored using different parameters. We repeated this calculation with a varying number of different features (from 40 to 300) in the feature selection and found that the classifier’s best performance was achieved at 160 features (Figure 2) Therefore, we selected 160 as the optimal size of the final feature space for the classification analysis (i.e., the threshold was set at 160). We used this threshold value because many studies have used the same method for establishing the threshold (Corbetta and Shulman, 2002; Dosenbach et al., 2010; Shen et al., 2010; Zeng et al., 2012). In addition, this procedure was also used to choose the optimal value for the parameter C for the SVM algorithm. We repeated this calculation with a range of different values (dimension: 2–20 and C: 0.005:0.05:2). Then, we identified the values when the classifier achieved the maximum GR. We identified the optimal C as 0.01, which is consistent with previous studies (Besga et al., 2012; Zeng et al., 2012). When using 160 features in the feature selection (Figure 2A) and *C* = 0.01 for the SVM, the classifier achieved maximum performance (GR: 72%; sensitivity: 76%; specificity: 68%; Figure 2B). Permutation tests revealed that the classifier successfully learned the relationship between the resting-state functional connectivity data and the pre-training/post-training class labels (*p* < 0.0001).

### Altered Resting-State Functional Connections after Training

**Figure 3:**
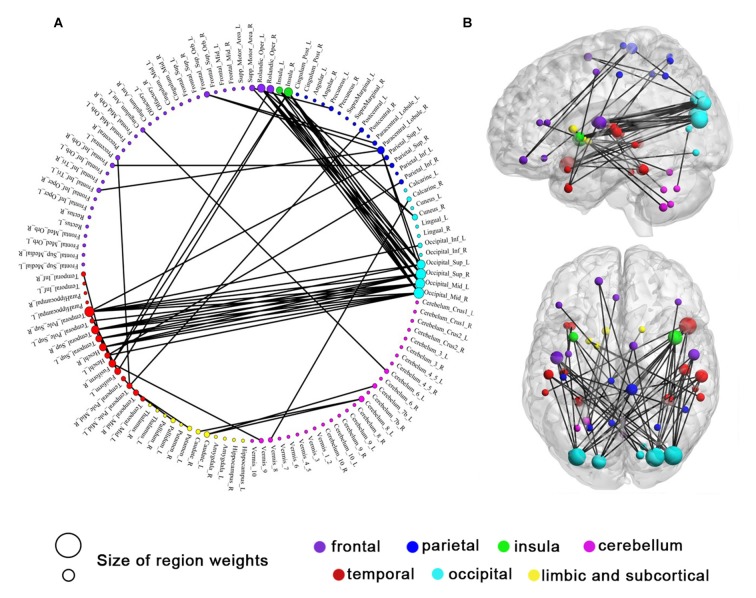
**Sixty consensus increased functional connections.** Regions are color-coded by category. The line colors represent the relative consensus functional connections. **(A)** Region weights and the distribution of consensus increased functional connections in a circle graph. **(B)** Consensus increased functional connections demonstrated in left sagittal, and top axial view. The colors represent structural categories of brain regions and the size of circles represent region weights.

Although 160 features were selected during a leave-one-out cross-validation iteration, the functional connectivity feature set selected in each iteration was slightly different (Dosenbach et al., 2010). In this investigation, 105 consensus functional connections were identified across the 50 (25 + 25 = 50) iterations of the cross-validation procedure (Dosenbach et al., 2010; Zeng et al., 2012). According to the Kendall tau rank correlation coefficient above, a positive correlation coefficient *τ_i_* represents the *i*th functional connectivity feature that exhibits a significant increase after IBMT training, while a negative correlation coefficient *τ_i_* represents the *i*th functional connectivity feature that exhibits a significant decrease after training. Comparing the consensus functional connectivity in subjects post-training vs. pre-training, we found more positive functional connections (60 connections) than negative connections (45 connections). This result indicates there are more increased functional connections after IBMT training. When analyzing the brain regions underlying this increase in functional connectivity, we found that occipital cortex (primarily including the superior and middle occipital gyrus) was functionally connected to many regions (Figure 3). Obviously, a large number of increased connections were encompassed between the occipital and temporal cortex (mainly comprising the superior temporal gyrus and its pole, and the insula), and between the occipital and the frontal cortex (mainly comprising frontal operculum). In addition, increased consensus functional connections between cerebellum and caudate were also detected (all *P* < 0.05). But we did not find significant lateralization differences among these bilateral areas.

## Discussions

Short-term mindfulness training induces a brain state that requires communication between multiple brain regions that collectively mediate the encoding and maintenance of sensory information (Tang et al., 2007, 2009, 2010, 2012, 2013, 2015a,b; Tang and Posner, 2014; Acevedo et al., 2016). Our results showed that 2 weeks of IBMT (5 h in total) reorganized the functional connectivity of large-scale brain networks involved in attention, cognitive and affective processing, awareness and sensory integration, and reward processing (e.g., the bilateral superior occipital/middle gyrus, bilateral frontal operculum, bilateral superior temporal gyrus, right superior temporal pole, bilateral insula, caudate and cerebellum.

Visual inputs contribute to over 90% of the total information (from all sensors) entering the brain. In literature, increased activity and connectivity in visual cortex are reported following short- and long-term mindfulness meditation (Tang et al., 2009, 2015a; Kilpatrick et al., 2011; Xu et al., 2014; Berkovich-Ohana et al., 2016). However, the underlying mechanism of visual cortex involvement during mindfulness remains unclear. One possibility might be that when meditators close eyes and focus on the inside world, the sensory processes are amplified. When they continuously observe the inner thoughts entwined with mental images, the mental processes of visual areas are heavily involved in. Another possibility might be the relaxation effect following meditation because the activity and functional connectivity of the visual cortex is also increased during light sleep, sedation and alcohol consumption (Kiviniemi et al., 2005; Horovitz et al., 2008; Esposito et al., 2010).

IBMT includes components of body relaxation, mental imagery and mindfulness (maintaining a high degree of awareness of body, mind and external instructions guided by an IBMT coach). One of our studies also detected greater activity in visual cortex following only five sessions of IBMT (Tang et al., 2009). It makes sense that the component of body relaxation and mental imagery could induce greater activity in visual areas, consistent with previous reports (Tang et al., 2009, 2015a; Kilpatrick et al., 2011; Xu et al., 2014; Berkovich-Ohana et al., 2016). However, mindfulness is different from sleep or sedation state with low level of arousal, and it requires to maintain high level of vigilance state for meditators. This is in line with our results that a large number of increased connections were encompassed between the occipital and temporal cortex (mainly comprising the superior temporal gyrus and its pole), and between the occipital and the frontal cortex (mainly comprising frontal operculum and insula).

Recent studies indicated that meditation modified subsystems of attention (Jha et al., 2007; Tang et al., 2007). It is worth mentioning that the frontal cortex participates both dorsal and ventral attention network (Petersen and Posner, 2012; Schmidt et al., 2013; Tang et al., 2015a). This network is believed to modulate externally directed attention by amplifying or attenuating the saliency of relevant and irrelevant cues (Corbetta and Shulman, 2002). It has been shown in the monkey that the combined actions of frontal eye fields and the occipital gyrus improved cross-area communication with attention (Gregoriou et al., 2009), and enhanced visual short-term memory performance (Liebe et al., 2012). In previous studies, we found that IBMT improves executive and altering attention networks compared to a well-controlled relaxation training (Tang et al., 2007, 2012, 2015a). Hence, we speculate that the long-range coupling between the occipital gyrus and frontal gyrus may improve and optimize global information processing helpful for the maintenance of a meditative state (Tang et al., 2007, 2015a; Tang and Posner, 2014).

Furthermore, we also found increased functional connectivity in adjacent occipital-temporal regions. These regions are often implicated in associative and item-recognition memory, a semantic network for both words and pictures, and self-cognition and awareness (Menon and Uddin, 2010). It might be possible that meditation training increases connections of temporal and occipital regions to allocate cognitive resources in order to improve performance. This idea is consistent with prior results showing that meditation improves attention and working memory performance (Tang et al., 2012, 2015a; Tang and Posner, 2014). The increased functional connectivity within temporal cortex is often associated with mood regulation and affective processing. Superior temporal sulcus was active in loving-kindness-compassion meditation (Lutz et al., 2008) and light modulation (Vandewalle et al., 2010). Insula was involved in interoceptive awareness, emotional responses and high-level attentional processes (Landtblom et al., 2011), consistent with our previous report that IBMT improves insula activity (Tang et al., 2009, 2015a). Importantly, using the Profile of Mood State and Attention Network Test, we found that IBMT improves attention and emotion regulation (Tang et al., 2007). The present results may indicate that IBMT improves emotion regulation through increased functional connectivity within temporal cortex.

In addition, increased consensus functional connections between cerebellum and caudate were also detected. Previous studies showed that the caudate nucleus plays a vital role in reward and learning, and the cerebellum may contribute to emotion and cognitive processing (Tang et al., 2009; Bostan et al., 2010; Ding et al., 2015, 2014). A recent study also showed that the basal ganglia and cerebellum may be linked together to form an integrated functional network that influences cognitive and affective processing (Bostan et al., 2010), and may support the brain state associated with meditation.

Taken together, our study indicates that MVPA of functional connectivity patterns in rsfMRI data effectively discriminates the different brain states in individuals before vs. after short-term meditation training. We found significantly increased functional connectivity between occipital, temporal and frontal regions, which may suggest that meditation training mainly improves attention, emotional, cognitive and reward processing. Our results provide new insights into the underlying neural mechanisms of mental training such as mindfulness, identifying complex changes in resting-state functional integration across the brain as a result of brief mindfulness training. It should be noted that we are aware of the potential issue of reverse inference when interpreting results (Poldrack, 2006). This is a legitimate first step in attempting to understand the significance of the observed changes in functional connectivity patterns following mindfulness training, and these results can be strengthened by future work focused on the functional selectivity and specificity of these changes in neural connectivity.

## Author Contributions

Y-YT designed and conducted research; YT, Y-YT, RT and JAL-P analyzed data and wrote the article. 

## Conflict of Interest Statement

The authors declare that the research was conducted in the absence of any commercial or financial relationships that could be construed as a potential conflict of interest.

## Footnotes

^1^www.fil.ion.ucl.ac.uk/spm

^2^http://www.restfmri.net

## References

[B1] Acevedo B. P. Pospos S. Lavretsky H. (2016). The neural mechanisms of meditative practices: novel approaches for healthy aging. Curr. Behav. Neurosci. Rep. 3, 328–339. 10.1007/s40473-016-0098-x 27909646 PMC5110576

[B2] Berkovich-Ohana A. Harel M. Hahamy A. Arieli A. Malach R. (2016). Alterations in task-induced activity and resting-state fluctuations in visual and DMN areas revealed in long-term meditators. Neuroimage 135, 125–134. 10.1016/j.neuroimage.2016.04.024 27109713

[B3] Besga A. Termenon M. Graña M. Echeveste J. Pérez J. M. Gonzalez-Pinto A. (2012). Discovering Alzheimer’s disease and bipolar disorder white matter effects building computer aided diagnostic systems on brain diffusion tensor imaging features. Neurosci. Lett. 520, 71–76. 10.1016/j.neulet.2012.05.033 22617636

[B4] Bostan A. C. Dum R. P. Strick P. L. (2010). The basal ganglia communicate with the cerebellum. Proc. Natl. Acad. Sci. U S A 107, 8452–8456. 10.1073/pnas.1000496107 20404184 PMC2889518

[B5] Corbetta M. Shulman G. L. (2002). Control of goal-directed and stimulus-driven attention in the brain. Nat. Rev. Neurosci. 3, 201–215. 10.1038/nrn755 11994752

[B6] De Martino F. Valente G. Staeren N. Ashburner J. Goebel R. Formisano E. (2008). Combining multivariate voxel selection and support vector machines for mapping and classification of fMRI spatial patterns. Neuroimage 43, 44–58. 10.1016/j.neuroimage.2008.06.037 18672070

[B7] Ding X. Tang Y. Y. Cao C. Deng Y. Wang Y. Xin X.. (2015). Short-term meditation modulates brain activity of insight evoked with solution cue. Soc. Cogn. Affect. Neurosci. 10, 43–49. 10.1093/scan/nsu032 24532700 PMC4994853

[B8] Ding X. Tang Y. Y. Tang R. Posner M. I. (2014). Improving creativity performance by short-term meditation. Behav. Brain Funct. 10:9. 10.1186/1744-9081-10-9 24645871 PMC3994657

[B9] Dosenbach N. U. Nardos B. Cohen A. L. Fair D. A. Power J. D. Church J. A.. (2010). Prediction of individual brain maturity using fMRI. Science 329, 1358–1361. 10.1126/science.1194144 20829489 PMC3135376

[B10] Esposito F. Pignataro G. Di Renzo G. Spinali A. Paccone A. Tedeschi G.. (2010). Alcohol increases spontaneous BOLD signal fluctuations in the visual network. Neuroimage 53, 534–543. 10.1016/j.neuroimage.2010.06.061 20600963

[B11] Fox M. D. Raichle M. E. (2007). Spontaneous fluctuations in brain activity observed with functional magnetic resonance imaging. Nat. Rev. Neurosci. 8, 700–711. 10.1038/nrn2201 17704812

[B12] Gregoriou G. G. Gotts S. J. Zhou H. Desimone R. (2009). High-frequency, long-range coupling between prefrontal and visual cortex during attention. Science 324, 1207–1210. 10.1126/science.1171402 19478185 PMC2849291

[B13] Guyon I. Elisseeff A. (2003). An introduction to variable and feature selection. J. Mach. Learn. Res. 3, 1157–1182.

[B14] Haxby J. V. (2012). Multivariate pattern analysis of fMRI: the early beginnings. Neuroimage 62, 852–855. 10.1016/j.neuroimage.2012.03.016 22425670 PMC3389290

[B15] Haynes J. D. Rees G. (2006). Decoding mental states from brain activity in humans. Nat. Rev. Neurosci. 7, 523–534. 10.1038/nrn1931 16791142

[B16] Horovitz S. G. Fukunaga M. de Zwart J. A. van Gelderen P. Fulton S. C. Balkin T. J.. (2008). Low frequency BOLD fluctuations during resting wakefulness and light sleep: a simultaneous EEG-fMRI study. Hum. Brain Mapp. 29, 671–682. 10.1002/hbm.20428 17598166 PMC6871022

[B17] Jha A. P. Krompinger J. Baime M. J. (2007). Mindfulness training modifies subsystems of attention. Cogn. Affect. Behav. Neurosci. 7, 109–119. 10.3758/cabn.7.2.109 17672382

[B18] Kendall M. Gibbons J. D. (1990). “Rank correlation methods edward arnold,” in A Division of Hodder and Stoughton, A Charles Griffin Title (London), 29–50.

[B19] Kilpatrick L. A. Suyenobu B. Y. Smith S. R. Bueller J. A. Goodman T. Creswell J. D.. (2011). Impact of mindfulness-based stress reduction training on intrinsic brain connectivity. Neuroimage 56, 290–298. 10.1016/j.neuroimage.2011.02.034 21334442 PMC3072791

[B20] Kiviniemi V. J. Haanpää H. Kantola J. H. Jauhiainen J. Vainionpää V. Alahuhta S.. (2005). Midazolam sedation increases fluctuation and synchrony of the resting brain BOLD signal. Magn. Reson. Imaging 23, 531–537. 10.1016/j.mri.2005.02.009 15919598

[B21] Kriegeskorte N. Simmons W. K. Bellgowan P. S. Baker C. I. (2009). Circular analysis in systems neuroscience: the dangers of double dipping. Nat. Neurosci. 12, 535–540. 10.1038/nn.2303 19396166 PMC2841687

[B22] Landtblom A. M. Lindehammar H. Karlsson H. Craig A. (2011). Insular cortex activation in a patient with “sensed presence”/ecstatic seizures. Epilepsy Behav. 20, 714–718. 10.1016/j.yebeh.2011.01.031 21440512

[B25] Lewis-Peacock J. A. Drysdale A. T. Oberauer K. Postle B. R. (2012). Neural evidence for a distinction between short-term memory and the focus of attention. J. Cogn. Neurosci. 24, 61–79. 10.1162/jocn_a_00140 21955164 PMC3222712

[B24] Lewis-Peacock J. A. Norman K. A. (2013). “Multi-voxel pattern analysis of fMRI data,” in The Cognitive Neurosciences, 4th Edn. eds Gazzaniga M. S. Mangun G. R. (Cambridge, MA: MIT Press), 911–920.

[B23] Lewis-Peacock J. A. Norman K. A. (2014). Competition between items in working memory leads to forgetting. Nat. Commun. 5:5768. 10.1038/ncomms6768 25519874 PMC4284654

[B26] Liebe S. Hoerzer G. M. Logothetis N. K. Rainer G. (2012). Theta coupling between V4 and prefrontal cortex predicts visual short-term memory performance. Nat. Neurosci. 15, 456–462. 10.1038/nn.3038 22286175

[B27] Lutz A. Brefczynski-Lewis J. Johnstone T. Davidson R. J. (2008). Regulation of the neural circuitry of emotion by compassion meditation: effects of meditative expertise. PLoS One 3:e1897. 10.1371/journal.pone.0001897 18365029 PMC2267490

[B28] Menon V. Uddin L. Q. (2010). Saliency, switching, attention and control: a network model of insula function. Brain Struct. Funct. 214, 655–667. 10.1007/s00429-010-0262-0 20512370 PMC2899886

[B29] Norman K. A. Polyn S. M. Detre G. J. Haxby J. V. (2006). Beyond mind-reading: multi-voxel pattern analysis of fMRI data. Trends Cogn. Sci. 10, 424–430. 10.1016/j.tics.2006.07.005 16899397

[B30] Pereira F. Mitchell T. Botvinick M. (2009). Machine learning classifiers and fMRI: a tutorial overview. Neuroimage 45, S199–S209. 10.1016/j.neuroimage.2008.11.007 19070668 PMC2892746

[B31] Petersen S. E. Posner M. I. (2012). The attention system of the human brain: 20 years after. Annu. Rev. Neurosci. 35, 73–89. 10.1146/annurev-neuro-062111-150525 22524787 PMC3413263

[B32] Poldrack R. A. (2006). Can cognitive processes be inferred from neuroimaging data? Trends Cogn. Sci. 10, 59–63. 10.1016/j.tics.2005.12.004 16406760

[B33] Satterthwaite T. D. Elliott M. A. Gerraty R. T. Ruparel K. Loughead J. Calkins M. E.. (2013). An improved framework for confound regression and filtering for control of motion artifact in the preprocessing of resting-state functional connectivity data. Neuroimage 64, 240–256. 10.1016/j.neuroimage.2012.08.052 22926292 PMC3811142

[B34] Schmahmann J. D. Doyon J. McDonald D. Holmes C. Lavoie K. Hurwitz A. S.. (1999). Three-dimensional MRI atlas of the human cerebellum in proportional stereotaxic space. Neuroimage 10, 233–260. 10.1006/nimg.1999.0459 10458940

[B35] Schmidt S. A. Akrofi K. Carpenter-Thompson J. R. Husain F. T. (2013). Default mode, dorsal attention and auditory resting state networks exhibit differential functional connectivity in tinnitus and hearing loss. PLoS One 8:e76488. 10.1371/journal.pone.0076488 24098513 PMC3788711

[B36] Shen H. Wang L. Liu Y. Hu D. (2010). Discriminative analysis of resting-state functional connectivity patterns of schizophrenia using low dimensional embedding of fMRI. Neuroimage 49, 3110–3121. 10.1016/j.neuroimage.2009.11.011 19931396

[B39] Tang Y. Y. Hölzel B. K. Posner M. I. (2015a). The neuroscience of mindfulness meditation. Nat. Rev. Neurosci. 16, 213–225. 10.1038/nrn3916 25783612

[B40] Tang Y. Y. Lu Q. Feng H. Tang R. Posner M. I. (2015b). Short-term meditation increases blood flow in anterior cingulate cortex and insula. Front. Psychol. 6:212. 10.3389/fpsyg.2015.00212 25767459 PMC4341506

[B43] Tang Y. Y. Lu Q. Geng X. Stein E. A. Yang Y. Posner M. I. (2010). Short-term meditation induces white matter changes in the anterior cingulate. Proc. Natl. Acad. Sci. U S A 107, 15649–15652. 10.1073/pnas.1011043107 20713717 PMC2932577

[B44] Tang Y. Y. Ma Y. Fan Y. Feng H. Wang J. Feng S.. (2009). Central and autonomic nervous system interaction is altered by short-term meditation. Proc. Natl. Acad. Sci. U S A 106, 8865–8870. 10.1073/pnas.0904031106 19451642 PMC2690030

[B37] Tang Y.-Y. Ma Y. Wang J. Fan Y. Feng S. Lu Q.. (2007). Short-term meditation training improves attention and self-regulation. Proc. Natl. Acad. Sci. U S A 104, 17152–17156. 10.1073/pnas.0707678104 17940025 PMC2040428

[B38] Tang Y.-Y. Posner M. I. (2014). Training brain networks and states. Trends Cogn. Sci. 18, 345–350. 10.1016/j.tics.2014.04.002 24816329

[B41] Tang Y. Y. Rothbart M. K. Posner M. I. (2012). Neural correlates of establishing, maintaining and switching brain states. Trends Cogn. Sci. 16, 330–337. 10.1016/j.tics.2012.05.001 22613871 PMC3419378

[B42] Tang Y. Y. Tang R. Posner M. I. (2013). Brief meditation training induces smoking reduction. Proc. Natl. Acad. Sci. U S A 110, 13971–13975. 10.1073/pnas.1311887110 23918376 PMC3752264

[B45] Tong F. Pratte M. S. (2012). Decoding patterns of human brain activity. Annu. Rev. Psychol. 63, 483–509. 10.1146/annurev-psych-120710-100412 21943172 PMC7869795

[B46] Vandewalle G. Schwartz S. Grandjean D. Wuillaume C. Balteau E. Degueldre C.. (2010). Spectral quality of light modulates emotional brain responses in humans. Proc. Natl. Acad. Sci. U S A 107, 19549–19554. 10.1073/pnas.1010180107 20974959 PMC2984196

[B47] Xu J. Vik A. Groote I. R. Lagopoulos J. Holen A. Ellingsen Ø.. (2014). Nondirective meditation activates default mode network and areas associated with memory retrieval and emotional processing. Front. Hum. Neurosci. 8:86. 10.3389/fnhum.2014.00086 24616684 PMC3935386

[B48] Zeng L. L. Shen H. Liu L. Wang L. Li B. Fang P.. (2012). Identifying major depression using whole-brain functional connectivity: a multivariate pattern analysis. Brain 135, 1498–1507. 10.1093/brain/aws059 22418737

